# Five decades of breeding populations census for 12 species of colonial waterbirds in northwestern Italy

**DOI:** 10.1038/s41597-023-02072-8

**Published:** 2023-04-26

**Authors:** Mauro Fasola, Daniele Pellitteri-Rosa, Guido Pinoli, Gianfranco Alessandria, Eleonora Boncompagni, Giovanni Boano, Anna Brangi, Franco Carpegna, Pietro Cassone, Mauro Della Toffola, Flavio Ferlini, Alessandra Gagliardi, Arturo Gargioni, Laura Gola, Nunzio Grattini, Marco Gustin, Franco Lavezzi, Lorenzo Maffezzoli, Cesare Martignoni, Roberto Musumeci, Giuliana Pirotta, Ivan Provini, Maurizio Ravasini, Alessandro Re, Bassano Riboni, Alberto Tamietti, Enrico Viganò, Michelangelo Morganti

**Affiliations:** 1 Dipartimento Scienze della Terra e dell’Ambiente, Università di Pavia, Via Ferrata 9, 27100 Pavia, Italy; 2 Regione Lombardia, Piazza Città di Lombardia 1, 20124 Milano, Italy; 3 Gruppo Piemontese Studi Ornitologici, Via San Francesco 188, 10022 Carmagnola, Italy; 4 Parco Ticino Lago Maggiore, Villa Picchetta, 28062 Cameri, Italy; 5 Dipartimento Scienze Teoriche e Applicate, Università dell’Insubria, Via Dunant 3, 21100 Varese, Italy; 6 Ente Gestione Aree Protette Po Piemontese, Lungo Po Gramsci 10, 15033 Casale Monferrato, Italy; 7 Lipu-BirdLife Italia, via Pasubio 3, 43121 Parma, Italy; 8 Parco Regionale Adda Sud, Viale Dalmazia 10, 26900 Lodi, Italy; 9 Parco Lombardo Valle del Ticino, Via Isonzo 1, 20013 Pontevecchio di Magenta, Italy; 10 Strada Rosa 108, 43011 Busseto, Italy; 11 CNR-IRSA, National Research Council - Water Research Institute, 20047 Brugherio, Italy; 12 Present Address: Istituto Oikos, Via Crescenzago 1, 20134 Milano, Italy

**Keywords:** Population dynamics, Conservation biology

## Abstract

Colonial waterbirds, a major biodiversity element occurring in the core of ultra-anthropized Europe, are ideal indicators of the wellness of inland wetlands. Nonetheless, there is a critical knowledge gap in their trend and population status. We present an uninterrupted 47 years-long dataset of the breeding populations of 12 species of colonial waterbirds (Ardeidae, Phalacrocoracidae, Plataleidae, Threskiornitidae) throughout a 58,000 km^2^ agricultural region in the higher Po basin (NW Italy). A trained team of collaborators censused with standardized field techniques the number of nests of each species at 419 colonies in the 1972–2018 period, summing up a total of 236,316 records. Data cleaning and standardization were performed for each census year, ensuring robust and consistent data. This dataset is among the largest ever collected for a guild of European vertebrates. It has already been used to describe the factors influencing population trends, and still offers opportunities to explore a wide range of key ecological processes such as biological invasions, global change consequences and biodiversity impact of agricultural practices.

## Background & Summary

Long-term datasets on animal populations are difficult to gather due to lack of commitment by researchers and to scarceness of funds that are necessary to sustain monitoring programs over many years^1–3^. A consequence of this is a systemic lack of long-term data that hinders the main mission of ecology, to understand how and why animal populations fluctuate. We contributed to filling this gap by carrying out a long-term monitoring of colonial waterbirds breeding in the same colonies (Ardeidae, Phalacrocoracidae, Plataleidae, Threskiornitidae) throughout a wide area in Northwestern Italy. Our monitoring program started in 1972 and is currently ongoing, thus reaching in 2022 the 51st year of uninterrupted counts and becoming the longest survey of bird populations in Italy and among the longest over whole Europe.

The monitored area is centered around the main rice-production zone of Europe, the plain of upper Po valley, that hosts one of the largest concentrations of breeding waterbirds at continental level^4^. Particularly abundant were the species of herons and egrets that during our monitoring fluctuated from 11,400 to 31,200 nests. At the peak of their abundance during our monitoring period, Grey Herons (*Ardea cinerea)* accounted for 7% of the mean number of pairs estimated for whole Europe^5^, Purple Herons (*Ardea purpurea)* for 7%, Squacco Herons (*Ardeola ralloides)* for 4%, while two species accounted for a very high proportion of the European breeding population, 29% in Little Egrets (*Egretta garzetta)* and 62% in Black-crowned Night-Herons (*Nycticorax nycticorax*).

These large populations were sustained by the availability of suitable foraging habitats, particularly rice fields that cover the largest area, host more abundant prey, and allow higher food intake for breeding herons and egrets, compared to the other European rice districts^6^. During the five decades of monitoring, the number of breeding waterbirds species increased from six to 12, including the allochthonous Sacred Ibis *Threskiornis aethiopicus*^7^, and their populations underwent large fluctuations. Some species of herons and egrets increased greatly from 1985 to 2000, thanks to reduced human-induced mortality and to meteorological variations^8,9^. But after 2000 the positive trend was reversed for the most abundant species, and in 2018 their number of nests had decreased by half in the sector of the monitored area where paddies were increasingly cultivated without permanent flooding, thus reducing the availability of foraging habitat. On the other hand, the same species remained stable or increased in the other two sectors where the main foraging habitats were rivers and ponds^4^. Wide surfaces cultivated on rice may act as surrogates of the nowadays lost natural wetlands for the freshwater biota, of which herons and egrets represent flagship species^10^. But the recent spread of rice cultivation practices that avoid permanent flooding^11^ threatens the pivotal ecological role of these crops for environmental conservation.

Subsets of this dataset have been repeatedly provided for free to researchers, to environmental managers, and to local government agencies. A notable result for nature conservation was that a considerable proportion of the colony sites have been progressively protected as nature reserves^12^.

Here, we make available to the scientific community a dataset on breeding distribution and nest numbers for 12 species of colonially breeding waterbirds, monitored over a large area along five decades. Population trends have been already analyzed, but the dataset still offers opportunities to explore a wide range of key ecological processes such as biological invasions, patterns of colony distribution, species interactions, influence of global changes, and impact of agricultural practices on biodiversity. The dataset is available in the LifeWatch ERIC metadata catalogue^13^. Currently, the dataset covers the first 47 years of monitoring, 1972–2018. The data for the years 2019–2022 have been collected but not yet completely verified. We look forward to produce updated versions of the dataset as soon as the quality checks will be concluded by the coordinating team.

## Methods

### Taxonomic coverage

We monitored the breeding population of 12 species, the seven species of the Family Ardeidae, and all the other waterbirds (Family Phalacrocoracidae, Plataleidae, Threskiornitidae) that bred in the same colonies throughout the study area. Until 1986, only five of these species were regularly breeding: Grey Heron, Purple Heron, Squacco Heron, Little Egret, and Black-crowned Night-Heron. Afterwards, five new species started breeding: Cattle Egret (*Bubulcus ibis)* since 1987, Great Cormorant (*Phalacrocorax carbo)* since 1989, Sacred Ibis since 1989, Great Egret (*Ardea alba)* since 1994, and Pygmy Cormorant (*Microcarbo pygmeus)* from 2014. Two additional species bred only sporadically and in low numbers: the Glossy Ibis (*Plegadis falcinellus)*, and the Eurasian Spoonbill (*Platalea leucorodia)*.

### Geographic coverage

**Fig. 1:**
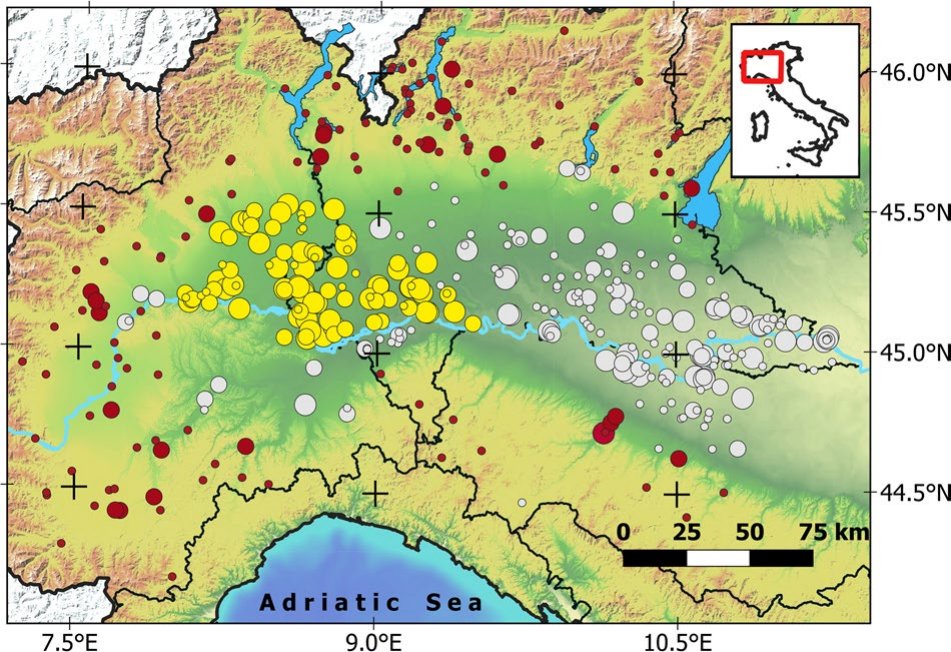
Study area in NW Italy where all the colony sites were monitored from 1972 to 2018. Dots colors group the colonies in the “Paddies” (yellow), in the “Rivers” (grey) and in the “Uplands” (red) sectors. Dot size classifies the colonies by the average number of nests over the entire monitoring period (small: <50 nests, medium: 51–250, large: >250). The hydrological network is visible on the map, with the main river crossing the area from west to east being the Po. Black contours represent Regional borders, white areas are non-Italian territories. Elevation model from^30^.

**Fig. 2:**
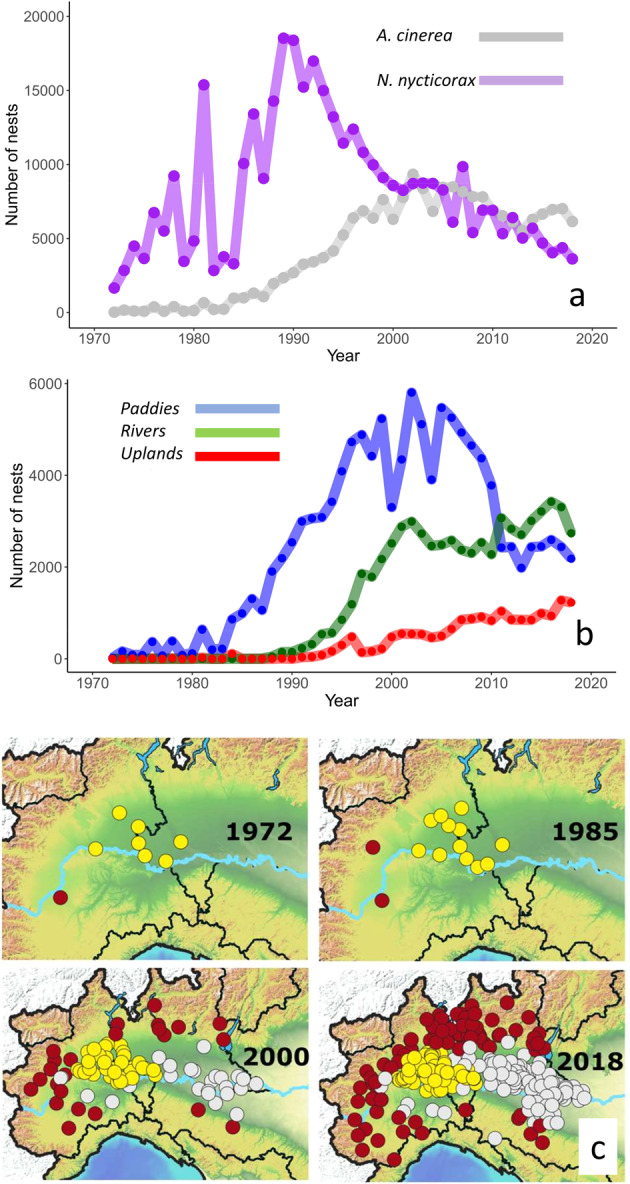
Examples of potentialities of the dataset. (**a**) comparison of population trends among Grey Herons (grey line) and Black-crowned Night-Herons (purple line). (**b**) comparison of population trends of *A. cinerea* over the three different sectors of the study area. (**c**) spatial expansion of Grey Herons over the study period; dots show the location of all the active colonies, and dot colors correspond to sectors as in Fig. 1.

**Fig. 3:**
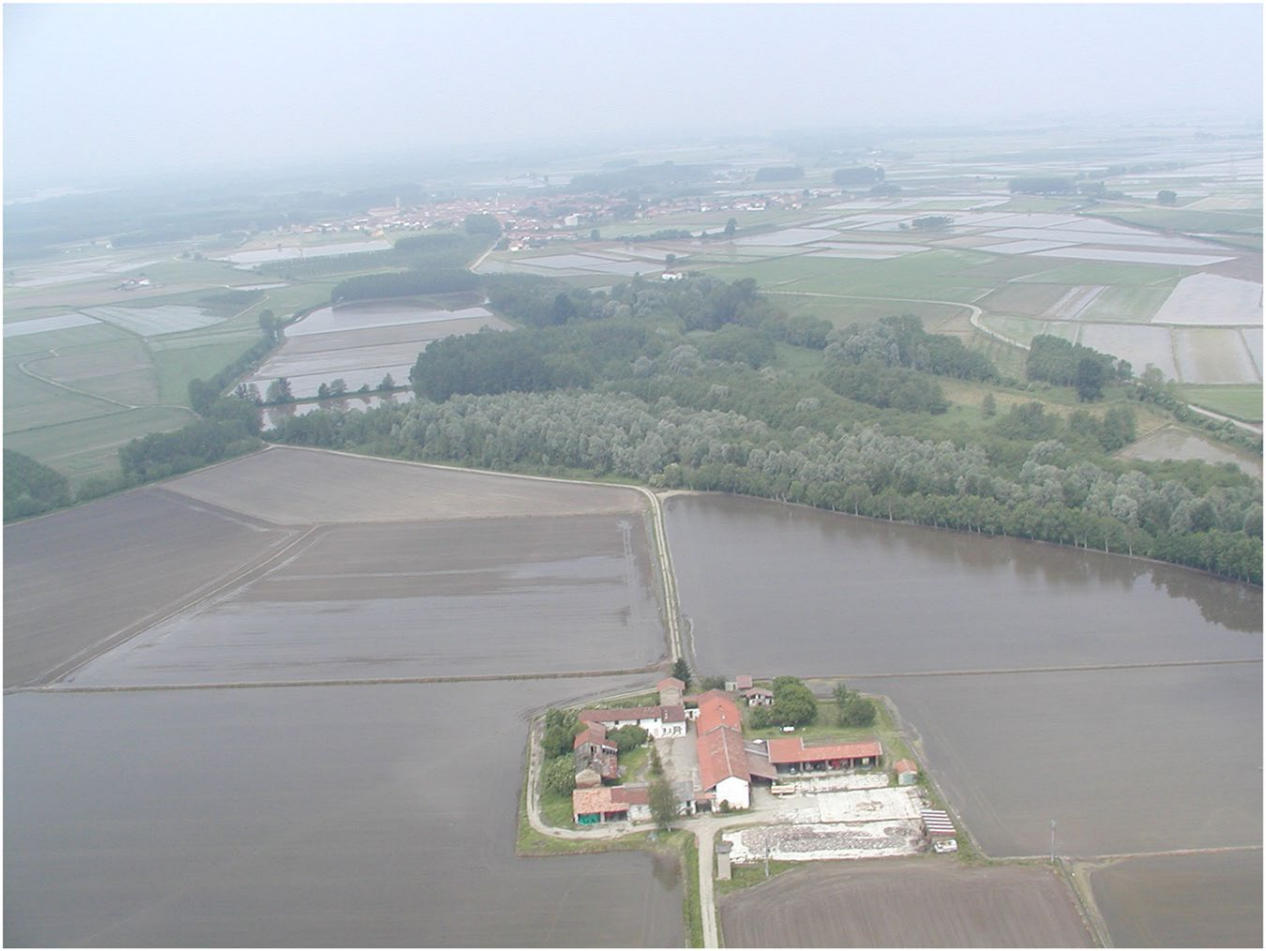
View of an area of intensive rice cultivation in in the ‘paddies’ sector. In 2001 all the cultivations were rice paddies with different levels of irrigation. The wood in the center hosted a mixed heronry during the whole period of monitoring and has been protected as Nature Reserve since 1985.

All the colony sites were monitored each year throughout 58,000 km^2^ in Northwestern Italy, the whole Lombardy and Piedmont regions, and the provinces of Piacenza, Parma, and Reggio Emilia (Fig. 1). This study area includes a variety of macro-habitats, wide cultivations and residual natural wetlands of the Po Plain, forests of the mid-altitude alpine valleys, and several villages and large urban areas such as Milan and Turin. The colonies recorded within the monitored area were classified into spatially contiguous groups, based on their location in one of three sectors: “Paddies”(category 1 in the dataset, column ‘Sector’), “Rivers” (category 2) and “Uplands”(category 3). This classification revealed in the years to be of high ecological meaning, since it condenses the fact that the three sectors differed in breeding species, in macro-ecological features, in species-specific patterns of range expansion (Fig. 2), in population trends^8^ and in the main foraging habitats and the prey types exploited by waterbirds^14^. Specifically, in the ‘paddies’ sector, located in the center of the study area below 200 m a.s.l., all the 12 species have bred and their populations have been more abundant than in the other sectors^9^. Here, herons and egrets foraged mainly on the extensive surfaces of paddies (Fig. 3), and on irrigation ditches, small wetlands and rivers, where they preyed mostly on amphibians, and recently also on allochthonous species, the crayfish *Procambarus clarkii* and the Oriental weatherfish *Misgurnus anguillicaudatus*^15^. In the ‘Rivers’ sector, located outside the rice cultivation area and below 250 m a.s.l., only some of the monitored species bred and with lower abundance, at least until 1990. Here, the banks of large rivers, Po, Ticino, Tanaro, Sesia, Adda, Oglio, and Mincio, represent the main foraging habitats and fish constitutes the main target prey. In the ‘Uplands’ sectors, located in the peripheral areas in hill and mountains landscapes from 250 to 800 m a.s.l., consistent breeding populations occurred only since 1990, mostly Grey Herons and a small number of other species (Fig. 2). Here, small streams, ponds and shallow lakes are the main foraging habitats for waterbirds^16^.

### Monitoring organization

The survey of the colonies and the nests counts were performed by teams of university researchers, officers of local administrations involved in nature conservation, park wardens, and voluntary collaborators, who had been initially trained during collective nest count sessions. The activities were organized throughout the whole period by the same coordinator (the first author here), who allocated the colonies to each collaborator, provided instructions and training aimed to homogenize the census techniques, and checked and archived the data.

The colony sites were initially detected from previous records, and by a thorough survey of the study area. Active colonies were generally easily spotted by observing the flights of the adults commuting from their nest to foraging areas, especially at peak breeding periods and in open plain landscapes. The exact location of the colony was then found by approaching and visiting accurately the area.

In this paper, we refer by the term “colony” to the whole of the breeding birds, their nests and the biotope where they are placed, by “colony site” to the biotope alone, and by “breeders” to the breeding birds alone. In most cases, the nests of a colony were tightly clumped and unequivocally segregated from neighbor colonies. Groups of nests distant at least 1 km were classified as distinct colonies, and higher distances were the norm. On the other hand, in a few cases we classified as a single colony the clumps of nests separated by spans of less than 1 km, because such distance allows visual or vocal interactions between the clumps, so that the breeders constitute a functional unit^17^. The number of nests per colony ranged from a few to thousands. The few cases of isolated nests that occurred especially for Grey Herons, were also archived as colonies. The breeders usually settled year after year in the same location, if the colony site remained suitable and undisturbed. In one notable case, Grey Herons have nested exactly at the same site in a suburban park at least since the year 1900 when this colony was first described^18^. However, at some of the monitored sites the breeders shifted location over short distances compared to previous years. As suggested by opportunistic observations, these movements may be due to habitat changes, or to human disturbance especially when this occurs during the initial phases of settlement when the breeders are highly sensitive. When a colony shifted location from one year to the next within a distance of less than 1 km and within the same patch of vegetation, we considered this as being the same colony. This rule conveniently avoided an unnecessary multiplication of colonies in the dataset. Even with this caution and the introduction of this spatial ‘threshold’, the cases of abandonment of a traditional colony site, and of settlement of a colony in a new site, were not rare. Consequently, the total number of colony sites recorded throughout the 47 years of monitoring (419) was much larger than the number of colonies active in a given year (from 45 in 1972 to 278 in 2018).

### Census techniques

**Fig. 4:**
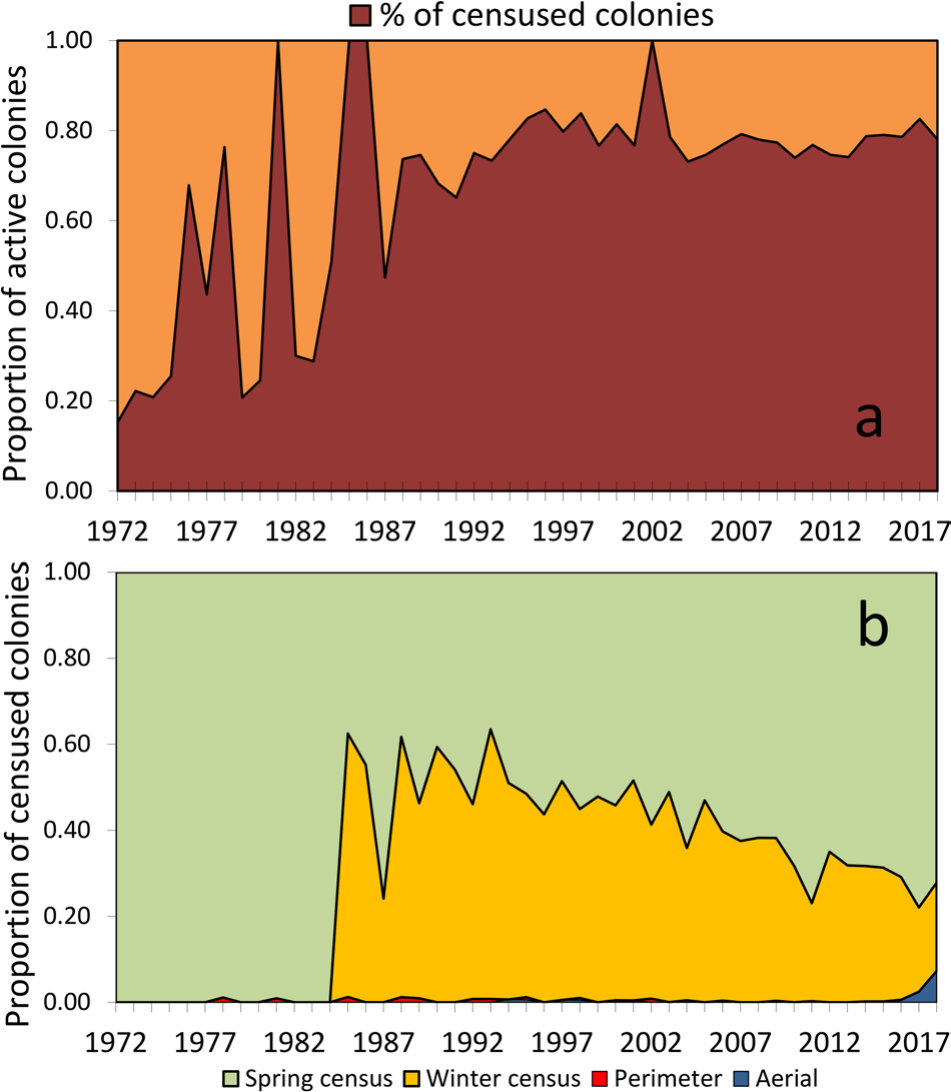
Proportion of colonies where the number of nests was estimated during the study period. (**a**) proportion of the active colonies where the number of nests was estimated (deep red), ad where only the occurrence of each species was reported (orange). Complete censuses (100% of active colonies exhaustively censused) were realized in the years 1981, 1985, 1986 and 2002. (**b**) yearly use of the different census techniques (see Methods) among the colonies in which nest counts were performed.

**Fig. 5:**
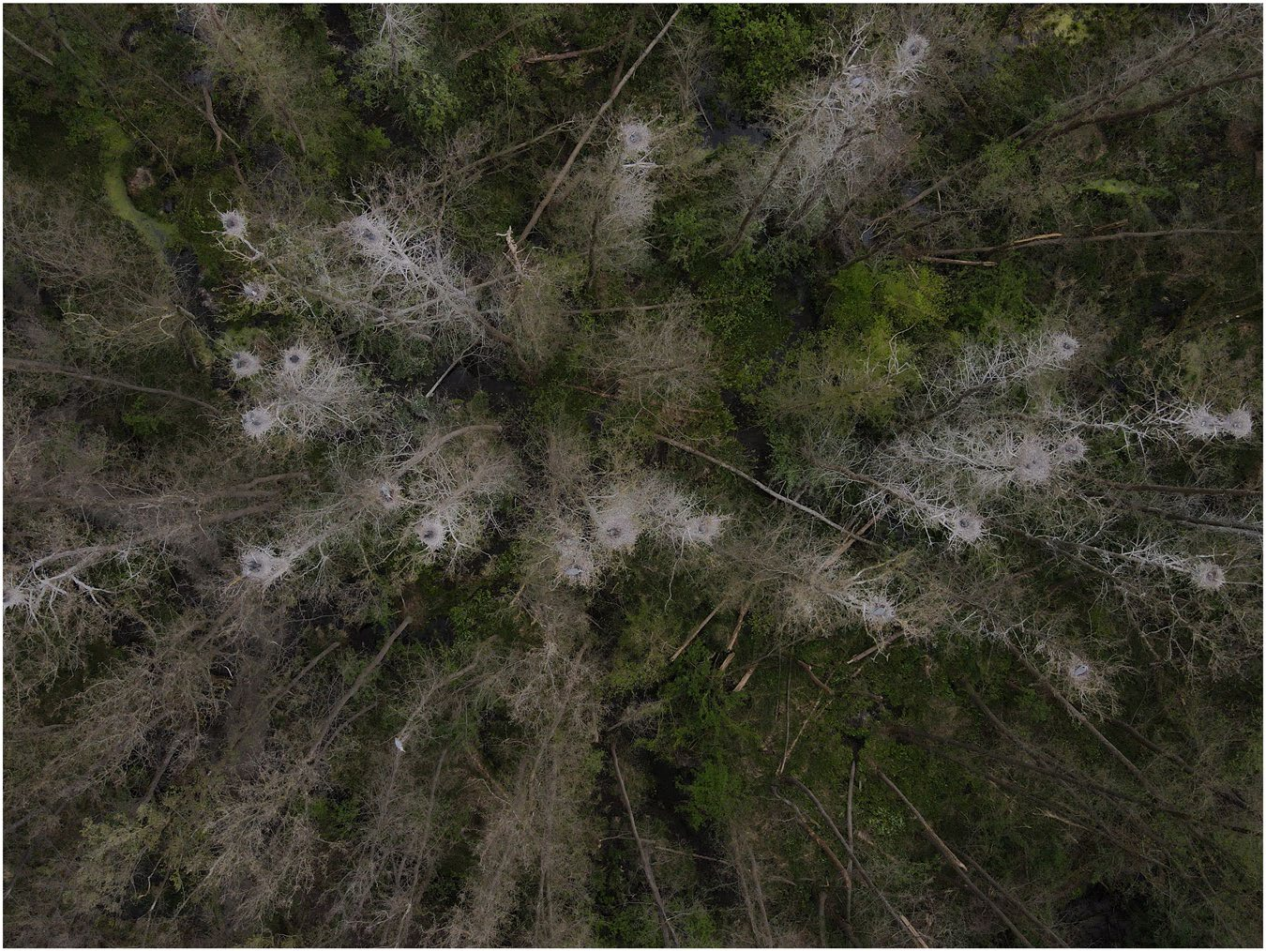
Example of an aerial photo used for nest counts. Monospecific colony with 25 nests of Grey Herons at egg hatching stage, on trees (Alder *Alnus glutinosa*).

The collaborators were instructed to visit each active colony at least two times during the breeding season, and to estimate the total number of nests of each species at the peak of their presence in the colony, using one of the following four standardized techniques that had already been proposed for Grey Herons^19^ (Fig. 4), each suited to differing habitats, colony size, and heron species, in order of decreasing preference. 1) Ground count of all the nests, performed at the peak occupation of each colony, a technique particularly suited for easily accessible, small (<100 nests), and monospecific heronries when birds were not severely disturbed by the observers. 2) Post-nesting ground count, a technique tested and advocated^20^ for Great Blue Herons *Ardea herodias*. The proportion of each species during their peak breeding season was estimated on a sample of nests during at least two visits. These proportions were then extrapolated to the total number of nests, counted during the subsequent autumn when the nests become clearly visible on leafless trees. Grey Heron nests were distinguished during post-breeding counts thanks to their larger size. The number of nests counted during fall was then multiplied by a conversion factor (1.12 for the Grey Heron, and 1.06 for the other species) that accounted for the average number of nests that had disappeared between the breeding period and the autumn count. These conversion factors had been estimated as the average ratio between total counts performed twice, during breeding and again during the subsequent autumn, at a sample of colonies (M. Fasola, unpublished data). Post-breeding nest counts were adopted particularly for large heronries with many Cattle Egrets, Little Egrets and Black-crowned Night-Herons, and when the disturbance caused by a complete count during the breeding period was considered excessive and not advisable. Even in these heronries, however, the scarce species, Purple Herons, Squacco Herons and Great Egrets, were counted individually during the peak breeding period, because estimated proportions during autumn are not proper in these less abundant species. 3) Aerial photographic count. Initially, nests were counted on low-altitude photos taken from ultralight aircrafts only in the few cases of colonies in reed beds. Since 2015, we increasingly used drones for aerial survey at several colonies in every sector of the study area, using a standardized procedure. At each colony, a sequence of contiguous, non-overlapping photos were taken by drones flown at 20–40 m above the nests. The number of nests was then counted on these photos by the same drone operator, with attention to include all the nests with incubating or brooding parents or with exposed eggs or chicks, to exclude birds not on their nest and non-breeding visitors, and to accurately identify the species within dense vegetation. The imagery taken by drones allows very accurate counts of all nests at sites with sparse foliage (Fig. 5). For colony sites in dense vegetation accuracy can be lower, therefore the estimates from aerial photos were checked by ground counts whenever the colony was accessible. Drones do not disturb the monitored species (personal observations) nor other species of colonial waterbirds^21^ during incubation or chick-rearing when flown at elevations >15 m above the nests. 4) Perimeter count, i.e., expert estimates based on the number of visible nests and foraging flights, observed from the colony edge. This technique was adopted in a few cases of small (<100 nests) and completely inaccessible heronries where other techniques were inapplicable.

The same census technique was normally adopted year after year for the same colony unless its size and characteristics changed and other census techniques were revealed to be more suited. The counting unit throughout the monitoring period was the number of nests that could be spotted in the active portion of the colony. In some cases, a minor proportion of nest structures from the previous year may persist in abandoned portions of the colony, but they were not included in the counts. On the other hand, we included in the counts all the visible nests within the active portions of the colony. We deemed it irrelevant to try and assess whether a nest was in use or not during the count, because observations of large samples of marked nests at several heronries, performed while recording breeding success^22^, showed that during the breeding season, abandoned nests within active portions of the colonies disappear rapidly as neighbour breeders steal their twigs. Egg development plus chick growth lasts about 90 days in the large Grey Heron and about 50 days in the small Squacco Heron^23^. The synchrony of breeding period and phases was usually high within each colony and breeding species, but could be variable among different colonies and particularly among certain species. Breeding occurred earlier for Grey Herons (with a peak from mid-February to mid-May), later for Purple Herons and for Squacco Herons (peak from mid-May to mid-July), and in a middle period (in April and May) for the other species. The phenological variations, both among heronries and species, were taken into account by checking the same colony during repeated visits and by performing the count of each species when the number of its nests was higher.

Nest counts were performed at more than 99% of the colonies in 1981, 1985, 1986, and 2002. In the other years, nest counts were accomplished at an average of 66.3% of the active colonies, and over the whole 1972–2018 period nest counts averaged 73.6% of the colonies (Fig. 4a). In the remaining cases, collaborators could not assess nest number for lack of time, fear of excessive disturbance to the breeders, site inaccessibility, or access denied by land owner. When nests could not be counted, breeding was confirmed for each species, colony and year, in order to provide the basic information allowing the calculation of a population index. Breeding confirmation without nest count occurred sporadically and randomly throughout all the study area and especially for the long-lasting colonies. This kind of incomplete count, requiring the adoption of population indexes, are typical of the censuses of colonial birds (e.g. herons in the UK^24^) and of most large-scale monitoring of environmental data. Abandoned colony sites were repeatedly checked as well, but reoccupation occurred only exceptionally.

## Data Records

The full dataset is publicly available at the LifeWatch ERIC data repository^13^ accessible from: 10.48372/bdc791a7-7678-44ad-a311-bd30c5086a06. This dataset includes a file “Waterbirds_Italy_1978_2018_V1.csv” with estimates of the number of nests for each of the 12 monitored species, at each of the 419 breeding sites, and for each of the 47 years from 1972 to 2018 (a total of 236,316 data). A metadata file describes the monitoring program, list the principal investigators, and defines the variables in the.csv file: geographic latitude and longitude of the geographic center of the locality of each colony site; sector of the study area; habitat of the colony site; year; scientific name of the species; occurrence status (whether absent or present and nesting); number of nests (or −1 when a species was recorded nesting but its number of nests were not assessed).

## Technical Validation

We are confident that virtually all the heronries within the study area had been identified, thanks to the re-occupation of traditional colony sites, to the repeated, independent discovery of the same heronries by different collaborators, and to the overall number of collaborators (over 100 in recent years). Only a few cases of isolated nests of Grey Herons may have been occasionally overlooked.

The nests counts were performed by collaborators, both professionals and amateur ornithologists, skilled in quantitative wildlife assessment. In order to homogenize the performance of the collaborators and to standardize the counts, we held training sessions, yearly meetings, and joint visits to a variety of colonies throughout the study area. It was not possible to evaluate the inter-observer differences in nest counts, and we are aware that despite the training, collaborators precision can be low^25^. Though the precision of the counts among the collaborators could not be determined, we are confident that the census data are robust and reasonably precise, because most of the sites have been visited by the same observer year after year, and in some cases throughout the whole study period. Inter-observer variability should therefore have had limited effect on the estimation of population trends, one of the main aims of this monitoring program.

We are aware of the shortcomings of the census techniques we employed. Such shortcomings, common to all censuses of colonially breeding ardeids, cormorants, and allied species, are well known and partly still unbridgeable^26^. Nest count accuracy is weakened by incomplete synchrony of breeding^27^, and by short nest persistence^28^, so that no practicable technique can count all the nests that existed throughout the breeding season. Therefore, neither the number of nests nor of breeders may be accurately estimated with the techniques currently available. This inaccuracy notwithstanding, the counts of breeding waterbirds can be used as indexes of population size^26^. Different census techniques provide estimates which have large confidence intervals (>20%^19^), and it is often impossible to compare the published estimates of colonial waterbirds, because the techniques for nest count are rarely described in any detail. However, thanks to our efforts to standardize the techniques adopted for the censuses in our study area, we are confident that our results can be interpreted as robust cues of nest numbers at peak breeding. Within these limits, we believe that the number of nest recorded during our standardized counts can be used for several aims, including the estimation of long-term temporal changes in breeding numbers.

## Usage Notes

### Advice on estimation of population trends using this dataset

The data of this monitoring program were used to assess the trends of the breeding populations over the past five decades, using the loglinear Poisson regression method as implemented by the TRIM software (https://www.cbs.nl/-/media/_pdf/2017/23/trim3man.pdf) and its R implementation (*rtrim* package^29^). These methods were specifically developed to analyze monitoring data from incomplete counts, which is the most commonplace in ecological surveys. TRIM software produces models of population trends, taking into account the typical overdispersion and temporal autocorrelation of such data. In case of interest in obtain population trends, we suggest entering into the operational dataset for TRIM only the colonies with nest numbers assessed for >50% of the years, because the software cannot develop models when incomplete counts are prevailing. Our repeated test confirm that the exclusion of a few incomplete cases has a limited effect on the resulting models, as suggested by the same TRIM developer (Van Strien pers. comm.). For further details on use of TRIM index for population trend analysis, as well as on examples of usage of part of this dataset see^4^,^8^.

## Data Availability

No custom code was used to generate or process the data described in the manuscript.
